# *Paenibacillus larvae* Phage Tripp Genome Has 378-Base-Pair Terminal Repeats

**DOI:** 10.1128/genomeA.01498-15

**Published:** 2016-01-07

**Authors:** J. Abraham, A.-C. Bousquet, E. Bruff, N. Carson, A. Clark, A. Connell, Z. Davis, J. Dums, C. Everington, A. Groth, N. Hawes, N. McArthur, C. McKenney, A. Oufkir, B. Pearce, S. Rampal, H. Rozier, J. Schaff, T. Slehria, S. Carson, E. S. Miller

**Affiliations:** Department of Plant & Microbial Biology, North Carolina State University, Raleigh, North Carolina, USA

## Abstract

*Paenibacillus larvae* bacteriophage Tripp was isolated from an American foulbrood diseased honey bee hive in North Carolina, USA. The 54,439-bp genome is 48.3% G+C, encodes 92 proteins, no tRNAs, and has 378-bp direct terminal repeats. It is currently unique in Genbank.

## GENOME ANNOUNCEMENT

Bacteriophage Tripp was isolated on *Paenibacillus larvae* strain ATCC 9545, a bacterium that causes American foulbrood disease (AFB) of honey bees (1). The 9545 host used is designated the type strain with the alternate designations of 846 [NRRL B-2605, http://www.atcc.org/]. Comb swab samples from the frame of an AFB diseased hive in Lincoln County, North Carolina, USA were incubated in brain heart infusion broth plus thiamine and glucose with *P. larvae* 9545. Clarified and filtered (0.22 µm) enrichment broth (30°C, 48 h) was plated in soft agar overlays with fresh *P. larvae* cells and plaques were identified. A phage Tripp plaque was purified three times and then amplified using the whole plate lysis method. DNA was extracted from the high titer lysate, and a sequencing library was prepared and analyzed using Illumina MiSeq procedures as described previously (2). FastQ files were assembled using CLC genomics workbench software (release 2014) with 10,071-fold coverage, and annotation was performed using DNA Master (http://cobamide2.bio.pitt.edu), GeneMark (3), NCBI BLASTp (4), ShineFind in the Galaxy bioinformatics suite (https://galaxyproject.org/), and HHPred (5). The 54,439-bp genome is 48.3% G+C, and encodes 92 proteins and no tRNAs.

The genome of *P. larvae* phage Tripp differs considerably from the genomes of other *Siphoviridae P. larvae*-infecting bacteriophages isolated in North Carolina (2) and from elsewhere in the world (6–8). While all other *P. larvae* phages to date are *cos* type with 5′ or 3′ overhanging ends, Tripp has terminal repeats of 378 bp. Tripp does show nucleotide and coding sequence (CDS) similarities to prophage sequences in the annotated *P. larvae* genome DSM 25430 (9) (GenBank accession number CP003355). Phage Tripp, although isolated as forming clear plaques, is likely a temperate phage with the capacity to form lysogens on certain *P. larvae* strains. Several biologically interesting properties of the Tripp genome were identified, including an encoded protein resembling the anti-toxin HicB, an anti-repressor, a transposase gene with an apparent −2 frameshift, and the terminally redundant direct repeats not present in the other deposited *P. larvae* bacteriophage genomes. One copy related to the repeat sequence (357/378 bp; 94%), occurs in the *P. larvae* DSM 25430 genome at base position 2,669,085 near other Tripp-like phage sequences. This suggests that strain DSM 25430 harbors a defective prophage lacking one of the terminal repeats.

Compared to the several Diva-like phages infecting *P. larvae* that have relatively short 5′- or 3′-overhanging DNA termini, the direct terminal repeats of the Tripp genome suggests it replicates and/or packages by a different mechanism. The growing number of sequenced phages that infect *P. larvae* provides increased opportunities for comparative phage genomics, new resources for genetic manipulation, and potential biotechnology applications involving AFB and other systems using *Paenibacillus* species.

### Nucleotide sequence accession number.

The complete genome of *Paenibacillus larvae* bacteriophage Tripp is available at Genbank under accession number KT755656.
